# The role of patients in pressure injury prevention: a survey of acute care patients

**DOI:** 10.1186/s12912-014-0041-y

**Published:** 2014-12-06

**Authors:** Elizabeth McInnes, Wendy Chaboyer, Edel Murray, Todd Allen, Peter Jones

**Affiliations:** Nursing Research Institute - St Vincents Health Australia, Sydney and Australian Catholic University (NRI), St Vincent’s Hospital, Level 5, deLacy Building, 390 Victoria Street, Darlinghurst, NSW 2010 Australia; School of Nursing, Midwifery and Paramedicine, Australian Catholic University, 33 Berry Street, North Sydney, NSW 2060 Australia; NHMRC National Centre for Research Excellence in Nursing (NCREN) Centre for Health Practice Innovation, Griffith Health Institute, Griffith University - Gold Coast, Gold Coast campus, Griffith University, QLD 4222 Australia; St Vincent’s Private Hospital, Sydney, Level 4, 406 Victoria St, Darlinghurst, NSW 2010 Australia; SVH Nursing Division Nurse Education & Development Centre, St Vincents Hospital, 390 Victoria Street, Darlinghurst, NSW 2010 Australia

**Keywords:** Patient views, Patient participation, Pressure injury prevention

## Abstract

**Background:**

Pressure injury prevention (PIP) is an important area of patient safety. Encouraging patient participation in care is a growing trend in healthcare as it can increase adherence to treatment plans and improve outcomes. Patients in acute care settings may be able to take on an active role in PIP. However, there is limited information on patients’ views of their perceived role in PIP. The aims of our study were to survey hospitalised patients’ views on a) their perceived roles in PIP and, b) factors that enable or inhibit patient participation in PIP strategies.

**Methods:**

Eligible participants were 18 years of age or older, from a neurology or orthopaedic ward and had been admitted to hospital at least 24 hours prior to enrolment in the study. A questionnaire comprising of fixed and open-ended responses was administered by researchers to participants. Numerical data was analysed descriptively and free-text comments were content-analysed and grouped into themes.

**Results:**

The mean age of participants (n = 51) was 65 years (sd = 16.6); over half were female and three quarters were orthopaedic surgical patients. Eighty-six per cent of participants understood the concept of pressure injury and 80% agreed that patients have a role in PIP. Participants nominated the following PIP strategies that could be undertaken by patients: *Keep skin healthy*; *Listen to your body* and *Looking after the inside*. Strategies required for patient participation in PIP were represented by three themes: *Manage pain and discomfort*; *Work together*; *Ongoing PI education*.

**Conclusion:**

To ensure successful participation in PIP, patients require education throughout admission, management of pain and discomfort and a supportive and collaborative relationship with health care staff. Health professionals should identify patient ability and motivation to prevent pressure injury (PI), work in partnership with patients to adhere to PIP, and ensure that PIP actions are facilitated with appropriate pain relief.

## Background

Pressure injuries (PIs) (also known as pressure sores, pressure ulcers or decubitus ulcers) are areas of localised damage affecting the skin and underlying tissue which result from pressure, and/or shear [1]. Hospital acquired pressure injuries (HAPI), that is those that originate in hospital, range from 6.4% - 17.4% of admissions [2-4]. PIs are painful and difficult to treat often necessitating surgery [5-7] and resulting in decreased physical function and quality of life [7,8]. They are also costly to the healthcare system. An Australian study of public hospitals predicted a median of 398,432 bed days lost, resulting in a median opportunity cost of AU$285 M as a result of pressure injuries [9].

In the light of the growing consensus that most hospital acquired pressure injuries are preventable and represent a significant patient safety issue [10], the pressure injury prevention (PIP) is a National Safety and Quality Health Service Standard in Australia [11] and has been referred to the UK National Institute of Clinical Excellence for development as a quality standard [12]. In the USA, Medicare hospitals no longer receive higher Medicare payments related to pressure injury related care of patients who acquire Stage III-IV PIs during their inpatient stay [13,14].

In response to these developments, healthcare services are increasingly focusing on the prevention and early detection of PIs [1,15]. Local and international guidelines recommend that patients at risk of PI, receive preventive measures including pressure-relieving or redistributing support surfaces and strategies such as turning and repositioning [15-17]. These PIP practices are important patient safety measures, but are time consuming, expensive, labour intensive and are less likely to be effective if patients are not fully engaged in their care [18].

There is also a growing trend to actively engage patients in participation in their healthcare [19]. Patient-centred models of care have been shown to enhance adherence to treatment, contribute to improved healthcare outcomes and positively increase patient safety in the acute care setting [20,21]. However, patient involvement in patient safety is dependent on a number of factors. For example, patients are less likely to initiate involvement if the task is complex [21], involves challenging a healthcare professional’s behaviour or requires adopting an unfamiliar action [22]. Moving and repositioning, hydration and skincare are key components of PIP [11]. These are neither complex concepts to understand nor difficult actions to undertake for some acute care patients. Hence, some patients may be able to assume a more active role in PIP in the hospital setting. This in turn could reduce some of the financial, time and physical burden associated with healthcare professional delivery of PIP and help to improve patient outcomes.

However, patients’ views of their perceived role in PIP is little understood [23,24]. A recent literature search undertaken for the purpose of this study found no articles exploring patients’ perceived role in PIP. Understanding how patients view their role and contribution to their PIP will provide an important insight into how patients can contribute to this important area of patient safety. The aims of our study were to survey acute care patients’ views on their perceived roles in PIP, and the factors that enable or inhibit their ability to undertake such a role.

## Method

### Design

A structured survey was administered to identify hospital patients’ understanding of PIP, their perceived role in PIP and the barriers and enablers to their participation in PIP strategies.

### Setting

The orthopaedic and neurology wards of two co-located metropolitan tertiary hospitals (four wards in total). Patients in these wards are at higher risk of pressure injuries due to impaired mobility from either surgery or having a neurological deficit [15]. Therefore sampling from these wards would provide valuable insights into what those at higher risk of PIs, perceive their role to be in PIP and the barriers and enablers to their participation in PIP.

### Sample and participants

A convenience sample of patients from the study wards was approached if they were 18 years of age or older, admitted to hospital at least 24 hours prior to administration of the survey, and were willing and able to consent to participation in the interviews. Patients not able to communicate verbally in English were excluded. If eligible patients were agreeable to participate in the study they were subsequently informed of the study procedure and processes and signed a consent form. If a patient declined, the next eligible patient was asked. Patients were entered into the study consecutively on the days of the week recruitment took place.

### Instrument

The questionnaire was developed and piloted by the researchers. The questionnaire was based on a review of patient safety and pressure injury literature [7,8,20,21,23] and national clinical guidelines and systematic reviews [11,16,17]. It consisted of five demographic questions and 18 fixed and multiple-choice items with provision for open responses, and five open-ended questions. Questions asked about: patients’ pain and comfort levels while in bed and when repositioning; their knowledge of PIs; their views on the patient role in PIP and also on the barriers and enables to patient participation in PIP. Following piloting with a sample of six patients, slight modifications were made to the wording of the questions. Data from the pilot were not included in the main study.

### Data collection

The questionnaire took between 10 and 15 minutes to administer. Each participant was interviewed at the bedside by one of two researchers, who were not employees of the hospital, during their hospital admission. Participants were given the answer options when questions were in the multiple choice format, and comments or short answer responses were recorded verbatim. The study was conducted between February and May 2012. Admission risk assessment scores and number of days post-operation were obtained from patient medical records. PI risk assessment was measured either by the Waterlow (Hospital 1) or Braden risk assessment tools (Hospital 2).

### Analysis

Closed response survey data were entered into SPSS^©^ version 19 (SPSS IBM Corporation, Armonk, NY, USA). Descriptive statistics were used to summarise data. Categorical data were reported as frequencies and percentages. Mean values and standard deviations were generated for continuous data. Free text responses were content-analysed and grouped into categories reflecting recurring themes relating to each open-ended question [25]. Themes were identified by examining regularities, convergences and divergences in the data. Some revisions were made to the themes following discussion amongst the investigators. Participant quotes were used to illustrate the themes.

### Ethical considerations

The study received ethics approval from the St Vincents Hospital (Sydney) Human Research Ethics Committee.

## Results

Sixty-three patients were approached to participate and from those 51 patients consented, giving a response rate of 80%. Demographic and pressure injury risk assessment details are provided in Table 1. The mean age of the participants was 65 years (sd = 16.6) and over half were female. Most participants were located on the orthopaedic ward and almost three quarters of the cohort were admitted as surgical patients (scheduled for or recovering from surgery). Approximately half of the sample were assessed as being at moderate-high risk of pressure injury as measured by the Waterlow or Braden tools. Four (8%) patients had a current PI (three were hospital acquired) and seven (14%) reported having had a PI within the past 12 months.

**Table 1:** **Participant demographics and pressure injury risk assessment** (***n*** = **51**)

	**Range mean (SD)**	**n**	**%**
Age	19 – 93; 65.0 (16.6)		
Sex			
Male		23	45
Female		28	55
Ward			
Orthopaedic		35	66
Neurology		16	34
Type of admission^			
Medical		13	26
Surgical		37	74
Number of days post-op	0 – 33; 10.1 (12.1)		
PI risk assessment^			
Waterlow not at risk		6	12
Waterlow at risk		5	10
Waterlow high risk		5	10
Waterlow very high risk		4	8
Braden no identified risk		13	26
Braden low risk		3	6
Braden moderate risk		2	4
Braden high risk		3	6
Risk assessment not documented		10	19

^Some missing data.

Most patients stated that they understood what a PI is (n = 41 80%) and agreed that patients had a role in PIP (n = 44 86%). Only a third (n = 19 37%) reported receiving PIP information from a staff member during their current admission.

When asked to comment on causes of pressure injuries, participants demonstrated some knowledge of the physiological risk factors for pressure injuries frequently citing ‘*reduced circulation*’ and immobility. One participant stated that a PI is something, ‘*they* [patients] *get if they are in hospital too long and definitely if you go to a nursing home*’.

Table 2 summarises the responses to questions about patient comfort. The mean pain score was 4.2 indicating moderate levels of pain. Approximately two-thirds of participants responded that thermal comfort level while in bed was ‘*just right*’. Over a half felt uncomfortable after an hour in bed. The main factors contributing to discomfort while sitting or lying in bed were being in one position, mattresses or cushions (75%). Comments included ‘*mattresses make back sweaty*’, ‘*mattress not large enough*’, ‘*terrible pillows and plastic coating makes me hot*’. More than half (51%) stated that being in one position while sitting or lying in bed contributed to discomfort. Discomfort was frequently cited as a motivating factor for self-repositioning. Over a half required assistance to reposition; the majority experienced no discomfort during repositioning.

**Table 2:** **Participant comfort** (**n** = **51**)

**Participant response**	**Range mean (SD)**	***n***	**%**
Pain score (range: 0 (no pain) -10 (high pain)	0 – 10; 4.2 (2.8)		
Temperature comfort in bed			
Too warm		9	18
Just right		34	67
Too cold		8	16
Comfort levels after 1 hour in bed			
Uncomfortable		27	53
Comfortable		24	47
Required assistance to change position (in bed or sitting)		31	61
Comfort during repositioning in bed without assistance			
Uncomfortable		12	24
Comfortable		39	77
Comfort during repositioning in bed with assistance			
Uncomfortable		3	10
Comfortable		28	90
Factors contributing to discomfort (lying or sitting)^#^			
Red, irritated skin		7	14
Wet or damp skin		7	14
Cushions		17	33
Mattresses		21	41
Being in one position		26	51

^#^Where totals add to >100%, more than one answer was possible.

Respondents identified a number of ways in which hospitalised patients could play a role in PIP. These strategies were grouped under three main themes as shown in Table 3: *Keep skin healthy*; *Listen to your body*; *Looking after the inside*. In relation to *Keep skin healthy* the majority of strategies included skin assessment; skin care (keeping skin moisturised and free of injury) and skin hygiene. Early intervention in the event of a developing PI was also regarded as important. *Listen to your body* included strategies focused on moving and repositioning; using equipment such as monkey-bars to help move self and also referred to the use of support and cushioning aids to reduce/relieve pressure. Some stated the importance of having access to ‘*special*’ mattresses and other support surfaces. There was also mention of strategies not supported by evidence, for example using ring cushions and the use of anticoagulation. Some participants stated that their skin was currently healthy and they were unconcerned with how to keep it healthy. *Looking after the inside* reflected views on the need to ensure that they ate well and kept hydrated.

**Table 3:** **Strategies participants identified for patient participation in PIP**

**Participant nominated PIP strategies**	
**Theme**	**Sub-themes with participant quotes**
Keep skin healthy	***Skin assessment***
	*Frequent skin assessment and early intervention*
	*Get regular skin checks from a specialist*
	***Skin care***
	*Keep clean and fresh*
	*Wash with provided soaps*
	*Dry properly*
	*Moisturise regularly*-*use sorbolene*
	*Rub or massage area*
	*Dressings on exposed or damaged areas*
	*Stop scratching*
	*Take care not to injure self*
	*Stop clothes bunching*
	*Sunshine*
	*Anti*-*coagulants*
Listen to your body	***Repositioning & movement***
	*Walk around the ward*
	*Turning and moving important* – *prevention better than cure*
	*Get up a bit more instead of using a bottle*
	*Aim to get moving as soon as possible*
	*Avoid lying down too long*
	*Keep up current activity* – *be more active and keep moving*
	*Get up and down*
	*Keep out of bed*
	*Change position while in bed*
	*Use monkey bar to reposition and to get comfortable*
	*Constantly move heels to relieve pressure*
	*Use side*-*rails to move*
	*Not sit in one spot all the time or for too long*
	*Reposition self as much as possible*
	***Use supports and cushioning aides***
	*Pillows under sore spots*
	*Pillows under calves to prop heels off bed*
	*Use supports to move hips to feel better*
	*Special mattresses*
	*Inflatable rings to sit on*
Looking after the inside	*Drink more*
	*Eat well*
	*Have enough to eat*
	*Keep hydrated*

Table 4 shows the three themes associated with barriers and enablers to patient participation in PIP: *Mange pain and discomfort*; *Work together*; and *Ongoing pressure injury education*. The first theme, *Manage pain and discomfort*, indicates physical factors such as pain, stiffness, numbness and a lack of strength affected ability to change position while in bed or in a chair. Some patients stated that the pain and effort required to move was a disincentive to unaided repositioning. In addition, the discomfort caused by mattresses, described as ‘hard, hot and sticky’, was an impediment to moving. Heavy bandaging or splinting of a limb made mobilisation and position changes problematic as did infusion pumps, in-dwelling catheters, drains, monitors and negative pressure wound therapy devices. Several patients also said that they lacked the confidence to mobilise (due to illness, injury or recent surgery).

**Table 4:** **Participant nominated strategies to facilitate patient participation in PIP** (**open**-**ended responses**)

**Strategies for patient participation in PIP**	**Enablers**	**Barriers**
	**(Illustrated by participant quotes)**	**(Illustrated by participant quotes)**
***Manage pain and discomfort***	*Nurses to check pain and comfort levels*- *we can*’*t move if uncomfortable*	***Patient factors***
		*Constant pain*, *stiffness*, *numbness* – *pain*, *the psychological barrier*
	*Confidence to move*	*Have not moved much because I*’*m in pain*
		*Don*’*t have strength to change and shift position*
		*Trying to get comfortable is hard because of my surgery*
		*Fairly helpless because can only use one arm*
		*Rheumatoid arthritis*
		*Risk of dislocation*
		*Sleeping on side is difficult*
		*Lack of confidence to move* (*due to illness*, *pain*, *injury*, *surgery*)
		***Equipment factors***
		*Hard*, *hot and sticky mattress*; *sinking in the middle therefore I need assistance to move*; *terrible pillows*; *mattress too small*
		*Afraid the monkey bar will break*.
		***Treatment factors***
		*Heavy splinting*, *bandaging*, *infusion pumps*, *IDCs*, *drains*, *monitors*, *attached to vac machine*; *negative pressure wound therapy*
		*All the buttons*, *wires and call buttons*
		*Knees are heavily bandaged in the straight position*
		*Painful cannula*
***Work together***	*Co*-*operation between patient and nurse*	*Waiting for staff*
	*Staff assistance to moisturise legs as unable to reach them*	*Reluctant to ask for help*
	*Work with nurses when time to be turned*	*Staff are too busy*
	*Call for assistance*	
	*Tell nurses when signs of PI appear*	
	*Regular checking and reminding by staff of things that should be done* (*eg reminders to move*)	
	*Motivate self*	
***Ongoing PI education***	*Verbal and written information during preadmission and hospital admission*	*Information too early in the patient journey*
	*Read all the information*	

In terms of the second theme, *Work together*, participants identified/highlighted the importance of involvement of staff, for example in prompting patients to move and checking patient comfort and pain levels. Requiring staff assistance and waiting for help from hospital staff were also nominated as barriers. Open-ended comments indicated some participants had not moved much in the past 24 hours, and others commented that they were rarely or never woken and encouraged to change position overnight. Respondents also expressed interest in working cooperatively with staff to prevent pressure injuries. *Ongoing PI education* both face to face and in the form of leaflets, was the third theme. While some suggested that information on PIP should be given during the preadmission consultation, others suggested in the post-operative period when they were on the path to recovery and be more likely to retain information. Others mentioned the importance of education throughout the admission period.

## Discussion

This study produces new knowledge on patients’ knowledge of pressure injury prevention , their views on their perceived roles in pressure injury prevention and of barriers and enablers to patient participation in pressure injury preventive strategies. Importantly, most participants thought that patients have a role in PIP. When asked, what patients could do to keep their skin healthy, the majority of participant responses focused on evidence-based strategies such as mobilizing and changing position and the importance of skincare and hydration. Recent national guidelines confirm the importance of these strategies [11]. Other important PIP strategies including appropriate nutrition and skin assessment were also, although less frequently, identified by participants. While there was high awareness of the importance of repositioning and moving and also of skin-care, the nomination of non-recommended strategies such as massage and rubbing and the use of ring cushions suggest that patients would benefit from evidence-based PIP education.

Discomfort that arises out of inert positioning is a primary stimulus for repositioning [16,26]. Hospitalised patients, however, may not always be able to respond to this stimulus if pain or disability, prevent them from doing so. In the present study, some patients stated that the pain associated with repositioning was enough to deter them from such activity. Pain as a barrier to moving and repositioning has been noted by others [5,27]. This is of concern in relation to this sample, who being from either orthopaedic or neurology wards were considered at-risk of pressure injury. We found, as have others, that patients are often reluctant to communicate that they are in pain and request assistance [5] despite being in considerable discomfort. In our sample the range of pain experienced was from nil pain to up to the maximum score. Health care staff may underestimate how much pain is experienced by patients [5] and national guidelines recommend that pain assessment should be a fundamental component of pressure area care [11]. If pain is eliminated or minimised, patients may be more likely to adhere to self-maintenance behaviours such as turning and repositioning. Staff may also underestimate the discomfort caused to patients by cushions and mattresses, which were also cited by many participants as an impediment to moving.

Even patients that exhibit positive health-seeking behaviour are at risk of inappropriate self-management if they are not provided with appropriate resources [23]. In our study, two-thirds of participants had not received information on PIP and were therefore reliant on past experiences and their own knowledge and assumptions about PIP. However, participants believed prevention is better than cure and that PIP education should begin at or before admission. Frequently, patients are given PIP information sheets during pre-admission clinic. However, this is given at a somewhat stressful time when patients are preparing for or being assessed for planned surgery. Reading such information may therefore not be a priority at this time. Patients might be more receptive to PIP education when they are out of the immediate post-operative stage and further into their admission, and are more alert and able to implement some strategies themselves. Throughout hospital admission patient needs for information varies over time [28]. Therefore giving the patient written and verbal information on more than one occasion may better meet their needs.

Participants also thought that frequent reminding by nurses during ward rounds to mobilise or reposition would be helpful and also give patients the opportunity to request analgesia. That some participants said it was difficult to request assistance with PIP or that they did not feel empowered to do so, suggests that a way forward may be partnerships with staff and a ward culture whereby patients are informed that they have a right to prompt and ask for assistance with skin care, repositioning or help with addressing the issue of adequate temperature control where hot and sticky beds may compromise the maintenance of a healthy micro-climate [29]. Written information can also help build relationships between staff and patients by making it easier for patients to ask or raise questions during encounters with health professionals [28].

Patient-centred models of care in acute care settings increase adherence to treatment and contribute to improved outcomes [20,21]. Pressure injury prevention (PIP) strategies such as repositioning, skin care, including avoiding skin extremes of heat and cold, and hydration [11,29] are relatively simple actions, that for those patients who are able, can undertake and thus have a more active role in PIP. However, patient adherence to prevention strategies is dependent on a complex interplay of factors but can be improved by a number of strategies. Ensuring that the patient is supported by their health care team; that they understand their condition and the need for prevention strategies; and assessing if they have the resources, motivation and ability to act in concordance with the prevention or treatment plan [30] are key in facilitating self-management activities.

### Strengths and limitations

This was a small convenience sample of participants, hence the results may not be generalisable. However, participants were selected from hospital wards (that is, the orthopaedic and neurological) that are considered to include those patients who are amongst the most at-risk of developing PIs and we were able to directly obtain their views on what they believe to be the patient’s role in pressure injury prevention. While the survey instrument was developed specifically for this study and no formal psychometric testing was done, the survey was piloted and standardised, and face-to-face administration of the survey by trained researchers ensured that there was no missing data.

## Conclusion

This study showed that although patients demonstrated a good awareness of the need to respond to signs and symptoms of potential pressure injury, patient knowledge of PIP was variable. Furthermore, their involvement in PIP was reliant on a number of factors such as having pain and discomfort addressed, feeling empowered to request assistance and the need for ongoing PIP education. In order to provide patient-centred care and ensure adherence to PIP regimes, healthcare professionals need to ensure that patients understand their risk of PI and how and why they are working toward prevention goals. Furthermore, healthcare professionals should identify the patient’s ability and motivation to prevent PI and ensure that they are adequately supported to adhere to prevention regimes, and that pain and comfort levels are managed to facilitate movement and repositioning. Future research on health professionals’ perspectives about patient participation in PIP is required.

## Abbreviations

PIP: Pressure injury prevention
PI: Pressure injury
PIs: Pressure injuries
